# Multi-Functional Ethylene-vinyl Acetate Copolymer Flexible Composite Film Embedded with Indium Acetate-Passivated Perovskite Quantum Dots

**DOI:** 10.3390/polym15193986

**Published:** 2023-10-04

**Authors:** Sheng Huang, Shasha Gao, Hui Zhang, Ce Bian, Yulong Zhao, Xiuquan Gu, Wenjie Xu

**Affiliations:** School of Materials Science and Physics, China University of Mining and Technology, Xuzhou 221116, Chinasdyulong@cumt.edu.cn (Y.Z.)

**Keywords:** CsPbBr_3_, perovskite, surface passivation, stability, WLED, solar cell

## Abstract

In recent years, all-inorganic cesium lead halide perovskite quantum dots have emerged as promising candidates for various optoelectronic applications, including sensors, light-emitting diodes, and solar cells, owing to their exceptional photoelectric properties. However, their commercial utilization has been limited by stability issues. In this study, we addressed this challenge by passivating the surface defects of CsPbBr_3_ quantum dots using indium acetate, a metal–organic compound. The resulting CsPbBr_3_ quantum dots exhibited not only high photoluminescence intensity, but also a remarkably narrow half-peak width of 19 nm. Furthermore, by embedding the CsPbBr_3_ quantum dots in ethylene-vinyl acetate, we achieved stretchability and significantly enhanced stability while preserving the original luminous intensity. The resulting composite film demonstrated the potential to improve the power conversion efficiency of crystalline silicon solar cells and enabled the creation of excellent white light-emitting diodes with coordinates of (0.33, 0.31). This co-passivation strategy, involving surface passivation and polymer packaging, provides a new idea for the practical application of CsPbBr_3_ quantum dots.

## 1. Introduction

Cesium lead halide perovskite quantum dots (QDs) exhibit excellent light absorption performance [1], a high photoluminescence quantum yield (PLQY) [2,3], a narrow emission half-peak width [4], a long carrier diffusion length [5], and an adjustable spectral range [6,7]. These desirable properties make them highly promising for applications in light-emitting diodes (LEDs) [8,9,10,11], solar cells [12,13,14], gas-sensing detection [15,16,17], photodetectors [18], and other fields [19,20,21]. However, the practical application of cesium lead halide perovskite quantum dots is hindered by their poor stability in light, water, oxygen, and heat [22,23,24,25]. This is primarily due to their ionic properties and low formation energy, which make them susceptible to ion migration and structural decomposition. Moreover, the dynamic nature of surface ligands can lead to their detachment from the quantum dots during purification and storage, resulting in the formation of surface defects and reduced colloidal stability [26,27,28,29]. Over time, these quantum dots are prone to decomposition and aggregation under storage, light, and heating conditions, ultimately impacting their photoelectric performance. To enhance the long-term stability of perovskite QDs, various strategies, including polymer packaging, surface defect reduction, and passivation techniques, have been employed [30,31,32,33]. Liu et al. made use of the high transparency, hydrophobicity, temperature resistance, and tensile properties of polydimethylsiloxane (PDMS) to prepare CsPbBr_3_@PDMS microspheres, improve the performance of QDs in water and acid-based environments, and apply them to a wearable field [34]. Shi et al. used ZrO_2_ and polystyrene (PS) to package CsPbBr_3_; after high-temperature heating and cooling cycles, the assembled white light-emitting diode (WLED) maintained its initial emission intensity, even during long-term high-power operations [35]. Ma et al. used the flexibility of EVA and the porosity of SiO_2_ to encapsulate CsPbBr_3_ so that the QDs were isolated from water and oxygen, and maintained a fluorescence intensity of 67.4% after 30 days in an indoor environment [36]. Shi et al. made use of the high flexibility of TPU; the CsPbBr_3_@Cs_4_PbBr_6_/TPU composite film maintained its initial luminous intensity when stretched to 900%, which expands the application range of QDs [37]. Lai et al. used the synergistic action of acid ions and metal ions in aluminum acetate to develop a novel double-emission CsPbBr_3_/Eu-MOF composite material with expected fluorescence intensity changes, which still had 50% luminescence intensity after heating at 100 °C for 3 h [38]. Song et al. developed a ZnO@CsPbBr_3_-Cs_4_PbBr_6_/EVA composite film, which used a ZnO coating on the surface of QDs and the characteristics of EVA as a battery-packaging material to improve the stability of QDs. After 30 days of indoor placement, the battery efficiency was basically the same as at the beginning [13]. Pan et al. employed a bidentate ligand to passivate CsPbI_3_ quantum dots post-synthesis. The red quantum dots produced using this passivation method exhibited a high external quantum efficiency (EQE) of up to 5.02%, a brightness of 748 cd/m^2^, and good stability [39]. Wang et al. employed 1-Octyl-3-methylimidazolium bromide to passivate CsPbBr_3_, creating a bromine-rich environment that prevented the formation of bromine defects on the perovskite surface, thereby enhancing the stability of the perovskite. The PLQY of the prepared CsPbBr_3_ reached as high as 90.93% [40]. The above studies indicate that both passivating agents and polymers can improve the stability of perovskite QDs. Nevertheless, the synergistic regulation of them is rarely reported.

Indium acetate is composed of a metal cation and an acid anion, and metal ions and acid ions are the classical passivating groups of QDs. EVA is a key packaging material for solar cells. Therefore, in this study, we employed indium acetate (In(C_2_H_3_O_2_)_3_) as a passivating agent for perovskite QDs and utilized the polymer ethylene-vinyl acetate (EVA) for packaging, aiming at enhancing the stability of QDs in harsh environments. The film (CsPbBr_3_-In/EVA) prepared through this approach demonstrated excellent flexibility, extensibility, and good stability, thereby expanding the potential applications of QDs. We further applied the CsPbBr_3_-In/EVA film to cover crystalline silicon solar cells, and the experimental results showed a 0.21% improvement in the power conversion efficiency (PCE). Additionally, we combined blue gallium nitride LEDs, the CsPbBr_3_-In/EVA film, and commercial red powder to achieve a wide color gamut spectrum. The resulting combination exhibited a color gamut coverage of 131.34% NTSC; under the Rec.2020 standard, it also reached 98.07%. Compared with traditional LEDs, the white light source produced through this strategy exhibited significant advancements in terms of color purity and color-rendering performance. These findings also suggest the great potential of the CsPbBr_3_-In/EVA film prepared in this study as a green light source for LED display applications. In general, this work underscores the importance of defect passivation through metal–organic compounds and the utilization of polymer encapsulation in the future applications of perovskite materials. These strategies hold promise for the achievement of enhanced stability and performance in various optoelectronic devices.

## 2. Materials and Methods

### 2.1. Materials

Cesium Bromide (CsBr, 99.5%, Macklin), lead bromide (PbBr_2_, 99.9%, Macklin), oleic acid (C_18_H_34_O_2_, OA, 85%, Aladdin), oleylamine (C_18_H_37_N, OLA, 80–90%, Macklin), *N*,*N*-dimethylformamide (C_3_H_7_NO, DMF, 99.5%, Aladdin), dimethyl sulfoxide (C_2_H_6_SO, DMSO, AR, Macklin), toluene (C_6_H_5_CH_3_, 99.5%, general reagent), methanol (CH_4_O, 99.5%, Macklin), Indium(III) acetate (In(C_2_H_3_O_2_)_3_, 99.99%, Leyan), 3-(*N*,*N*-dimethyloctadecylammonio) propanesulfonate (C_23_H_49_NO_3_S, DMST, 97%, Aladdin), EVA ((CH_2_CH_2_)_x_[CH_2_CH(O_2_CCH_3_)]_y_, FIRST), red-emitting K_2_SiF_6_:Mn^4+^ (K_2_SiF_6_:Mn^4+^, KSF, ZKHY-C680A, ZhongKeHaoYe), and polyethylene terephthalate (PET, 100 × 100 × 1 mm, XuEr) were used.

### 2.2. Preparation of Precursor and EVA Solution

The CsPbBr_3_ precursor comprised 0.0426 g CsBr, 0.0734 g PbBr_2_, 0.023 g C_23_H_49_NO_3_S, 4 mL DMF, 1 mL DMSO, 0.5 mL OA, and 0.25 mL OLA. These were added to a 10 mL glass vial and stirred at 70 °C until a clear and homogeneous solution was obtained.

For the CsPbBr_3_-In precursor we added 0.01, 0.02, 0.03, 0.04, and 0.05 mmol In(C_2_H_3_O_2_)_3_ to the CsPbBr_3_ precursor, respectively, and dissolved by heating at 70 °C and stirring.

For the EVA solution, 1.2 g EVA and 4 mL toluene were added to a 10 mL glass vial and stirred to obtain a clear homogeneous solution at 70 °C.

### 2.3. Synthesis of CsPbBr_3_-In/EVA Film

**Figure 1:**
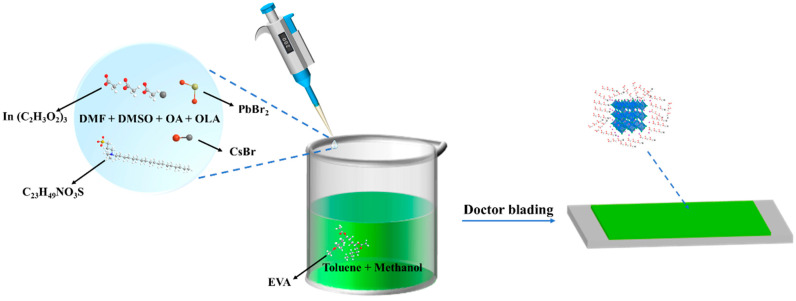
Synthesis process of CsPbBr_3_-In/EVA film.

We collected 20 μL of the precursor and added it to a mixture of 2 mL toluene and 20 μL methanol. The solution was stirred at room temperature, resulting in the formation of a CsPbBr_3_-In QD solution. Subsequently, we mixed the CsPbBr_3_-In QD solution with the EVA solution and stirred it at room temperature, leading to the formation of the CsPbBr_3_-In/EVA solution. At this point, the mass ratio of QDs to EVA was 0.034%. Finally, the solution was uniformly deposited onto a PET substrate and allowed to dry naturally at room temperature, resulting in the formation of a CsPbBr_3_-In/EVA film as illustrated in Figure 1.

### 2.4. Material Characterization

An X-ray diffractometer (XRD, D8 Advance, Bruker), field-emission scanning electron microscopy (FESEM, Hitachi New Generation SU8220, Tokyo, Japan) equipped with X-ray energy dispersive spectroscopy (EDS), and X-ray photoelectron spectroscopy (XPS, EscaLab 250Xi, Thermo Scientific, Waltham, MA, USA) with an Al Kɑ source and transmission electron microscopy (TEM, Tecnai G2 F20, FEI, Hillsborough, OR, USA) were used to study the structure, elemental composition, surface morphology, and microscopic morphology of the compound. The optical properties of the QD solution were characterized using a UV–Vis spectrophotometer (UV–Vis, Cary 300, Varian, Hong Kong, China). PL spectra (PL, FS5, Edinburgh, Scotland) were used to investigate the fluorescence characteristics of the perovskite quantum dot solutions and composite films. The carrier lifetime of the perovskite quantum dots was recorded by transient time-resolved photoluminescence (TRPL, FLS 980, Edinburgh, Scotland). The J-V curve of the electrochemical workstation (Keithley 2420 Source Meter, Hong Kong, China) was used to measure the photovoltaic performance of the silicon solar cell under a standard AM 1.5 simulated sun (100 mW·cm^−2^, Oriel Sol 3A, Newport, Irvine, CA, USA).

## 3. Results and Discussion

**Figure 2:**
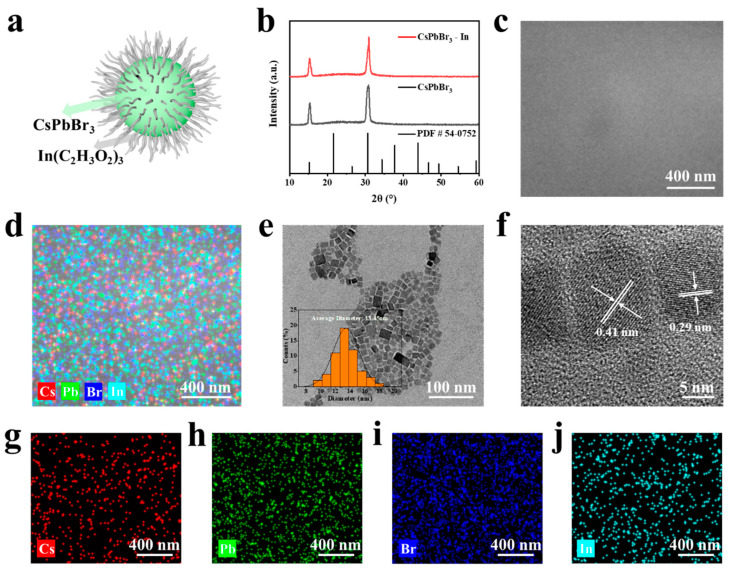
Structure characterization of CsPbBr_3_-In. (**a**) Diagram of CsPbBr_3_-In QDs. (**b**) XRD patterns of CsPbBr_3_-In QDs (red) and CsPbBr_3_ (black). The bottom data are the standard XRD cards of CsPbBr_3_. (**c**) SEM images of CsPbBr_3_-In/EVA film. (**d**) Elemental mapping images corresponding with (**c**). (**e**) TEM and (**f**) HRTEM images of CsPbBr_3_-In QDs, respectively. (**g**–**j**) Elemental mappings of Cs, Pb, Br, and In elements in the CsPbBr_3_-In/EVA film of Figure 2d.

The structure of CsPbBr_3_-In is depicted in Figure 2a. Initially, an XRD analysis was performed to characterize the synthesized sample, as depicted in Figure 2b. A comparison with the PDF standard card (54-0752) [41] revealed that CsPbBr_3_ and CsPbBr_3_-In shared the same crystal structure (space group Pm$\bar{3}$m, lattice constant a = b = c = 5.83 Å, α = γ = β = 90°). Notably, two prominent diffraction peaks at 15.18° and 30.64° corresponded with the (100) and (200) crystal faces of the cubic perovskite structure. The results showed that the QDs had a cubic perovskite structure and a preferred orientation of (100) [42]. Importantly, the addition of In(C_2_H_3_O_2_)_3_ did not lead to a shift in the diffraction peaks or alter the crystal structure of CsPbBr_3_, indicating that In^3+^ was not incorporated into the crystal structure of CsPbBr_3_-In. The SEM and EDS images are presented in Figure 2c,d, respectively. The observed sample surface appeared smooth and flat, with an even distribution of Cs, Pb, Br, and In elements. Further evidence of the uniform distribution of these elements (such as In) was obtained through the element mapping of Cs, Pb, Br, and In in single CsPbBr_3_-In QDs, as shown in Figure 2g–j, illustrating the effective coverage of indium acetate ligands on the perovskite QDs. Subsequently, TEM and HRTEM were employed for the further characterization of the obtained samples. As shown in Figure 1e, the TEM image revealed a significant number of uniformly dispersed CsPbBr_3_-In rectangular squares. A size distribution analysis of the CsPbBr_3_-In QDs indicated an average size of 13.45 nm, as shown in the illustration in Figure 2e. In Figure 2f, the measured distances between the crystal faces of CsPbBr_3_-In were found to be 0.41 nm and 0.29 nm, corresponding with the (110) and (200) crystal faces, respectively [43,44]. This further validated the XRD results, supporting the conclusion that the addition of In(C_2_H_3_O_2_)_3_ did not affect the crystal structure.

**Figure 3:**
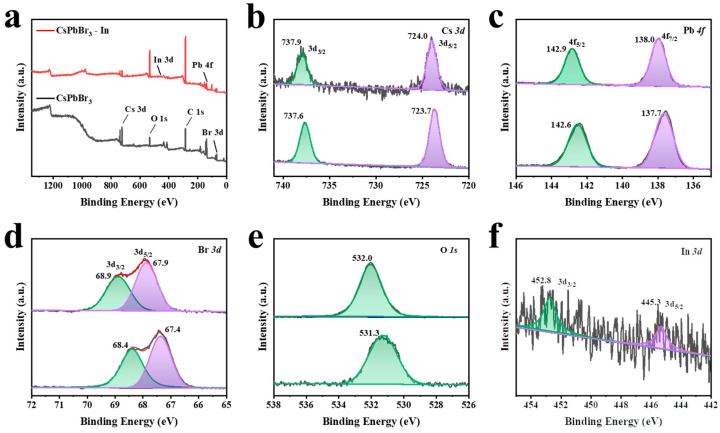
XPS characterization. (**a**) XPS spectra of CsPbBr_3_ before and after In(C_2_H_3_O_2_)_3_ treatment. XPS map of (**b**) Cs *3d*, (**c**) Pb *4f*, (**d**) Br *3d*, (**e**) O *1s*, and (**f**) In *3d*. The bottom images in (**b**–**e**) are CsPbBr_3_ QDs, and the upper of (**b**–**e**) are XPS spectra in CsPbBr_3_-In QDs.

X-ray photoelectron spectroscopy (XPS) was employed to investigate the surface composition and chemical state of the CsPbBr_3_ and CsPbBr_3_-In samples. Figure 3a displays the full-scan map of the two samples, revealing the presence of Cs, Pb, Br, C, and O elements; additionally, the CsPbBr_3_-In sample exhibited element In. As depicted in Figure 3b–d, the incorporation of In(C_2_H_3_O_2_)_3_ induced a shift in the binding energy of Cs *3d*, Pb *4f*, and Br *3d* peaks towards higher values compared with CsPbBr_3_. Previous literature has suggested that the movement of Pb *4f* and Br *3d* peak positions towards higher binding energy is beneficial, improving the stability of QDs [45]. Furthermore, Figure 3e shows a shift in the O *1s* binding energy of CsPbBr_3_-In from 531.3 eV to 532.0 eV, potentially indicating the formation of In-O bonds. The additional peaks of In in CsPbBr_3_-In, as depicted in Figure 3f, further corroborated the successful adsorption of In(C_2_H_3_O_2_)_3_.

**Figure 4:**
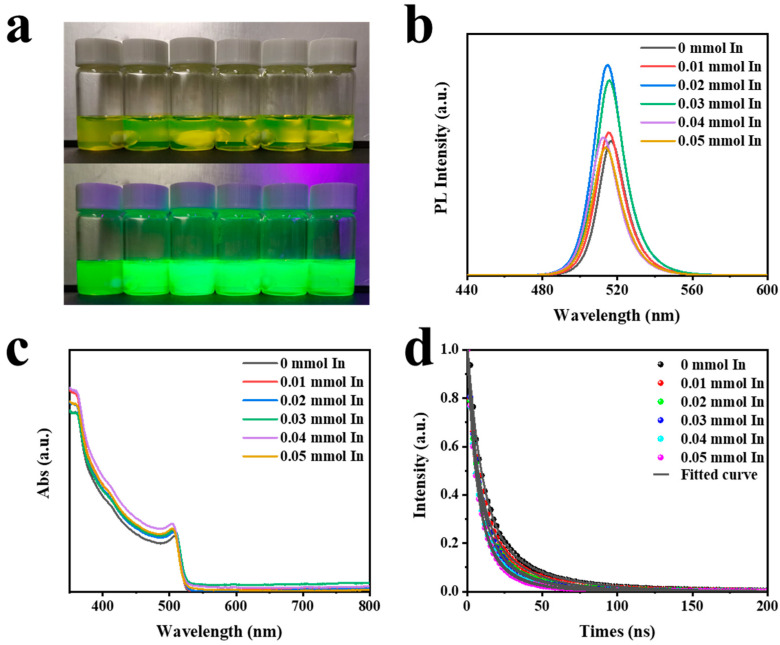
(**a**) Photos of perovskite quantum solutions with different contents of In(C_2_H_3_O_2_)_3_ under natural light and UV light. (**b**) PL, (**c**) UV–Vis, and (**d**) TRPL spectra of CsPbBr_3_ QDs with different contents of In(C_2_H_3_O_2_)_3_.

QD solutions with varying concentrations of In(C_2_H_3_O_2_)_3_ are shown in Figure 4a. The upper image was taken in an indoor environment, while the lower image was captured under UV light. Figure 4b displays the PL spectra corresponding with the different contents of In(C_2_H_3_O_2_)_3_ shown in Figure 4a. It was obvious that the addition of In(C_2_H_3_O_2_)_3_ had an impact on both the luminous intensity and peak position of CsPbBr_3_. Specifically, when 0.02 mmol In(C_2_H_3_O_2_)_3_ was added, the luminous intensity increased by a factor of 1.56. Therefore, in the follow-up test, we chose a precursor solution with a content of 0.02 mmol In(C_2_H_3_O_2_)_3_. However, excessive amounts of In(C_2_H_3_O_2_)_3_ led to a decrease in the luminescence intensity of CsPbBr_3_, accompanied by a blue shift in the peak position. This observation aligned with the UV–Vis absorption spectrum displayed in Figure 4c. Then, TRPL measurements were conducted on samples with varying concentrations of In(C_2_H_3_O_2_)_3_, as depicted in Figure 4d. The photoluminescence decay curves for both CsPbBr_3_ and CsPbBr_3_-In followed a double exponential pattern. The decay time, decay component ratio, and average decay lifetime are shown in Table 1. The longer-lived component (τ_2_) of CsPbBr_3_ could be attributed to typical radiative recombination, whereas the shorter-lived component (τ_1_) was likely associated with non-radiative recombination processes [46,47]. The average lifetime of perovskite quantum dots (QDs) with different concentrations of In(C_2_H_3_O_2_)_3_, as shown in Figure 4a, were reported to be 25.96, 21.16, 18.23, 17.15, 14.49, and 13.65 ns, respectively. The shorter decay lifetime was not indicative of more severe non-radiative recombination but rather faster carrier-radiative recombination, as non-radiative recombination would enhance the PL intensity [13]. This finding aligned with the higher PL observed in CsPbBr_3_-In. Hence, based on the TRPL results, it could be inferred that the addition of In(C_2_H_3_O_2_)_3_ was conductive to reducing defects, promoting faster carrier-radiative recombination, and enhancing luminescence.

**Figure 5:**
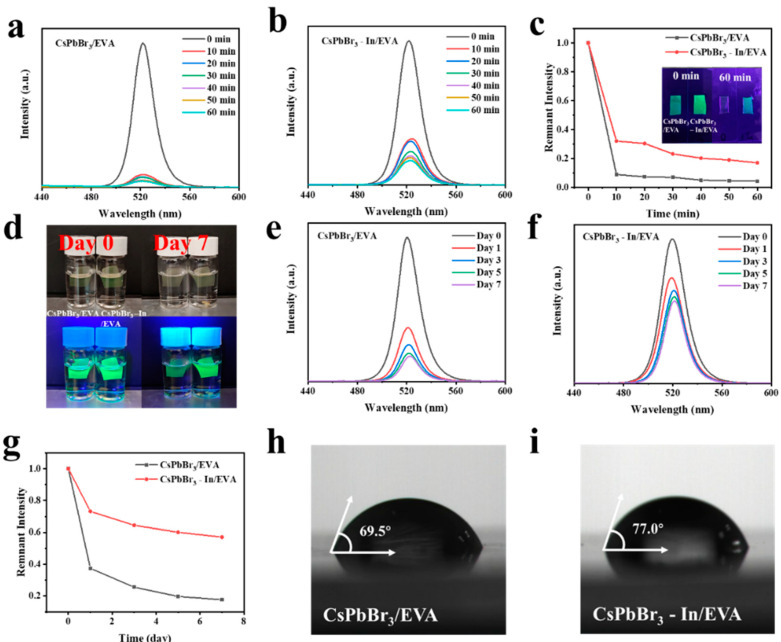
(**a**–**c**) Heat stability tests of CsPbBr_3_/EVA and CsPbBr_3_-In/EVA films. (**d**) Photos of perovskite quantum dot films under natural and UV light before and after immersion in water. (**e**–**g**) Water stability tests of CsPbBr_3_/EVA and CsPbBr_3_-In/EVA films. Contact angle tests of (**h**) CsPbBr_3_/EVA and (**i**) CsPbBr_3_-In/EVA films.

In order to further passivate the defects and improve the stability of the QDs, we encapsulated the CsPbBr_3_ and CsPbBr_3_-In QDs in EVA and investigated the stability of the CsPbBr_3_/EVA and CsPbBr_3_-In/EVA films under extreme conditions, including exposure to water and high temperatures. The results of the stability tests at 90 °C are presented in Figure 5a–c. It was evident that the addition of In(C_2_H_3_O_2_)_3_ significantly enhanced the thermal stability. Subsequently, we immersed the films in water and conducted tests, as shown in Figure 5d–g. It was observed that both the CsPbBr_3_/EVA and CsPbBr_3_-In/EVA films experienced substantial degradation on the first day. However, after 7 days, the CsPbBr_3_-In/EVA films maintained a good luminous performance, while the CsPbBr_3_/EVA films exhibited more significant deterioration. This improvement in stability may be attributed to the passivation of QD defects and enhanced stability resulting from the presence of In(C_2_H_3_O_2_)_3_. Furthermore, we performed a contact angle test (Figure 5h,i), which revealed that the contact angle increased by 7.5° upon the addition of indium. Consequently, the perovskite film exhibited enhanced hydrophobicity. Therefore, the inclusion of In(C_2_H_3_O_2_)_3_ has the potential to improve the stability of perovskite films in water and high-temperature environments. This enhancement in stability further expands the application range of quantum dots (QDs) in various fields.

**Figure 6:**
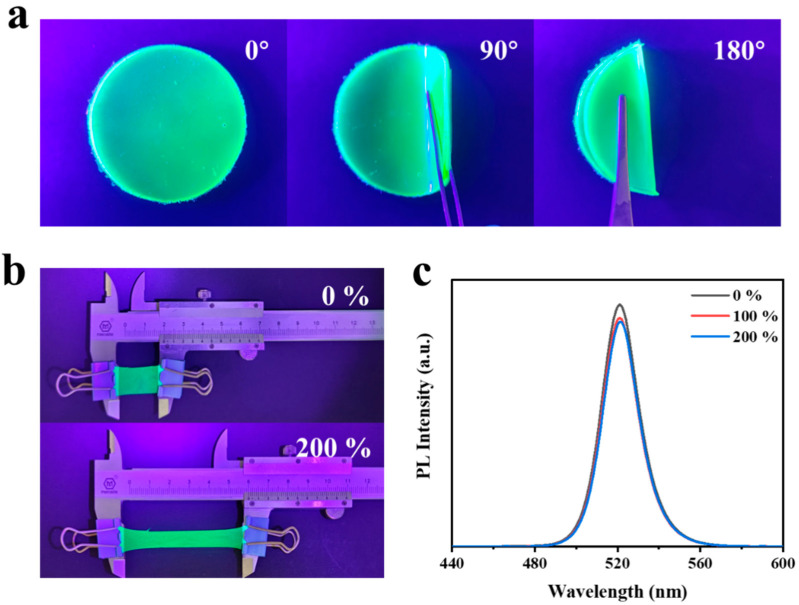
(**a**) CsPbBr_3_-In/EVA film with different bending angles under UV lamp. (**b**,**c**) Stretch stability tests of CsPbBr_3_-In/EVA film.

With the development of flexible solar cells and displays, the flexibility of QD films has attracted more and more attention. Therefore, the flexibility of the film is then further tested [48]. As shown in Figure 6a, the flexibility of CsPbBr_3_-In/EVA films was demonstrated when bent at different angles. The film exhibited excellent flexibility, allowing it to be easily shaped into various forms. To assess its performance under stretching, the PL strength measurement was obtained before and after stretching (see Figure 6b,c), and the following stretching equation was adopted to calibrate its performance:

(1) $$\sigma =\frac{L_{h}-L_{0}}{L_{0}}\times 100\%$$

where L_0_ and L_h_ represent the initial and final lengths of the film, respectively. During the tensile test, the CsPbBr_3_-In/EVA film was initially 2 cm in length and did not break, even when subjected to a stretching rate ranging from 0% to 200%. This exceptional performance was primarily attributed to the stretchability of the EVA polymer. By assessing the PL at different stretching rates, there existed only a slight decrease in the luminescence intensity of the CsPbBr_3_-In/EVA film before and after stretching, which indicated that the use of EVA packaging enabled the QDs to exhibit flexible stretchability, thus expanding the potential applications of QD films in various fields. In addition, we also investigated the effects of different amounts of EVA polymers on the bending and stretching properties, as shown in Figures S1 and S2. When the mass ratio of CsPbBr_3_-In and EVA was 0.023%, although the film had good tensile and bending properties, the flatness of the film was poor and the quantum dots aggregated. When the mass ratio of CsPbBr_3_-In to EVA was 0.045%, the surface of the sample was relatively flat and there was no obvious aggregation phenomenon of quantum dots, but the bending and tensile properties were poor. We theorized that the reason for this phenomenon was when there was less EVA, there was not a good link between the polymer chains, resulting in poor film formation. When there was more EVA, the force between the molecular chains increased and the stretching effect became worse.

**Figure 7:**
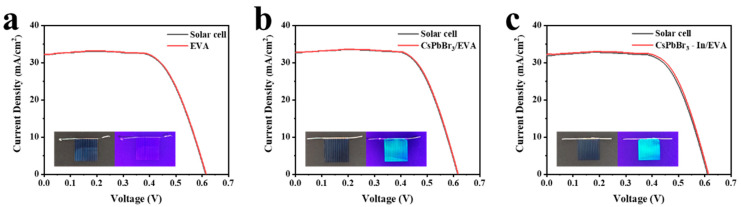
J-V curves of silicon solar cells using (**a**) EVA, (**b**) CsPbBr_3_/EVA, and (**c**) CsPbBr_3_-In/EVA.

In order to verify the photovoltaic performance after the film coverage, we tested the UV-visible absorption spectra of the EVA, CsPbBr_3_/EVA, and CsPbBr_3_-In/EVA films (Figure S3a). After adding indium acetate, the absorption performance improved to a certain extent. Subsequently, the EVA, CsPbBr_3_/EVA, and CsPbBr_3_-In/EVA films were deposited onto the surface of a silicon solar cell, as depicted in Figure 7a–c. Upon exposure to UV light, both the CsPbBr_3_/EVA and CsPbBr_3_-In/EVA films exhibited intense green luminescence. A rigorous comparison of the silicon solar cell’s performance was performed before and after applying the respective polymer films. The corresponding J-V curve of the silicon solar cell is shown in Figure 7a–c, and Table 2 provides the photovoltaic parameters. The results showed that the power conversion efficiency (PCE) improvements were 0.10%, 0.14%, and 0.31% for the devices incorporating EVA, CsPbBr_3_/EVA, and CsPbBr_3_-In/EVA, respectively. In addition, in order to further verify the impact of CsPbBr_3_-In/EVA on the efficiency of the battery, we also tested the J-V curve under ultraviolet light, as shown in Figure S3b–d and Table S1. Due to the weak ultraviolet light, the battery efficiency was low. Therefore, we used the following equation to evaluate the relative improvement in battery efficiency, E_R_:

$$E_{R}=\frac{E_{1}-E_{0}}{E_{0}}\times 100\%$$

where E_0_ and E_1_ represent the initial cell efficiency and the packaged battery efficiency, respectively. Through the calculation, we found that after EVA, CsPbBr_3_/EVA, and CsPbBr_3_-In/EVA packaging, the battery efficiency relatively increased by 10%, 22%, and 37%, respectively. The underlying mechanisms responsible for the enhanced device performance of EVA, CsPbBr_3_/EVA, and CsPbBr_3_-In/EVA were distinct; the improvement in EVA mainly stemmed from its ability to mitigate light reflection on the silicon solar cell’s surface, leading to an increase in the short-circuit current (J_SC_) [49]. In the case of the CsPbBr_3_/EVA and CsPbBr_3_-In/EVA composite films, the J_SC_ enhancement could be attributed to two factors. Firstly, similar to the EVA film, the composite films exhibited an anti-reflection effect, facilitating greater light transmission into the silicon solar cell. Secondly, the incorporation of CsPbBr_3_ quantum dots (QDs) into these films allowed for the conversion of low-efficiency, high-energy photons into more efficient, lower-energy photons [13].

**Figure 8:**
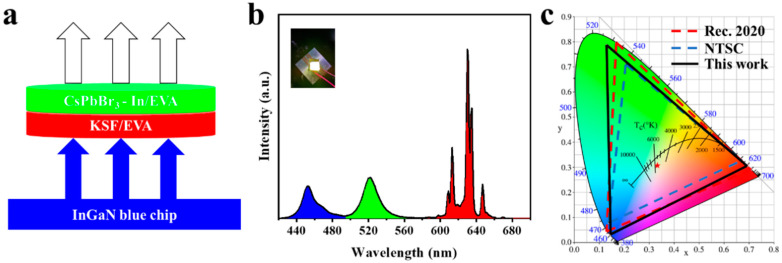
(**a**) Schematic diagram of WLED for the InGaN blue chip, KSF/EVA, and CsPbBr_3_-In/EVA, (**b**) PL spectrum of WLED (the picture attached is white light), (**c**) and CIE chromaticity coordinates of WLED.

Next, a white light-emitting diode (WLED) was developed by integrating a green light-emitting CsPbBr_3_-In/EVA film, a KSF red phosphor, and an InGaN blue chip (Figure 8a), aiming to demonstrate its potential applications in the displays. As shown in Figure 8b, the PL spectrum exhibited clear separation into three distinct regions—blue, green, and red emissions—corresponding with the blue LED chip, CsPbBr_3_-In/EVA film, and KSF phosphor, respectively. Without optimizing the device structure, the resulting WLED achieved color coordinates of (0.33, 0.31), which corresponded with 131.34% NTSC and 98.07% Rec. 2020 color gamut coverage, as depicted in the CIE coordination in Figure 8c. This suggests that the CsPbBr_3_-In/EVA film holds significant potential as a green light source for LED display devices.

## 4. Conclusions

In order to improve the stability of perovskite quantum dots and expand their application range, this work presents a straightforward synthesis method for CsPbBr_3_ QDs that effectively passivated QD defects by incorporating In(C_2_H_3_O_2_)_3_. When the content of In(C_2_H_3_O_2_)_3_ in the precursor was 0.02 mmol, the PL intensity was the highest. Subsequently, a co-passivation strategy was further adopted and the CsPbBr_3_-In QDs were encapsulated with the polymer EVA. When the mass ratio of QDs to EVA polymer was 0.034%, the CsPbBr_3_-In/EVA had excellent tensile and bending properties, thereby improving the stability of perovskite films in challenging environments and broadening their potential applications. To further enhance the performance of silicon solar cells, the CsPbBr_3_-In/EVA film was applied as a cover, effectively boosting the PCE of the cell. Lastly, the development of a WLED device that combined a blue chip, KSF red phosphor, and CsPbBr_3_-In/EVA film resulted in a WLED device with a wide color gamut for high-quality display applications, also exhibiting significant potential.

## Figures and Tables

**Table 1:** The fitted results of TRPL of CsPbBr_3_ with different In(C_2_H_3_O_2_)_3_ contents.

Sample	τ_1_ (ns)	A_1_	τ_2_ (ns)	A_2_	τ_A_ (ns)
0 mmol In	9.37	0.71	36.41	0.29	25.96
0.01 mmol In	6.85	0.62	27.07	0.38	21.16
0.02 mmol In	6.58	0.67	24.57	0.33	18.23
0.03 mmol In	7.30	0.70	24.11	0.30	17.15
0.04 mmol In	6.03	0.69	20.13	0.31	14.49
0.05 mmol In	5.98	0.71	19.43	0.29	13.65

**Table 2:** Photovoltaic parameters of devices with EVA, CsPbBr_3_/EVA, and CsPbBr_3_-In/EVA composite films.

Figure	Sample	Voc (V)	J_SC_ (mA/cm^2^)	Factor	Efficiency	ΔE
a	Solar cell	0.61296384	32.15262214	66.9669	13.1981	0.0975
	EVA	0.61512954	32.21299025	67.0983	13.2956	
b	Solar cell	0.61570937	32.69598568	68.3807	13.7659	0.1352
	CsPbBr_3_/EVA	0.61768150	32.71814702	68.7855	13.9011	
c	Solar cell	0.61018635	31.92773015	68.1391	13.2748	0.3111
	CsPbBr_3_-In/EVA	0.61358988	32.21761684	68.7256	13.5859	

## Data Availability

The data presented in this study are available on request from the corresponding author.

## Supplementary Materials

The following supporting information can be downloaded at: https://www.mdpi.com/article/10.3390/polym15193986/s1, Multi-Functional Ethylene-vinyl Acetate Copolymer Flexible Composite Film Embedded with Indium Acetate-Passivated Perovskite Quantum Dots.
