# Supplementary material for: Multi-Functional Ethylene-vinyl Acetate Copolymer Flexible Composite Film Embedded with Indium Acetate-Passivated Perovskite Quantum Dots
Source: Polymers (Basel). 2023 Oct 4;15(19):3986. doi: 10.3390/polym15193986 (PMC10575095; doi:10.3390/polym15193986)
Supplement: Supplementary file 1 [file polymers-15-03986-s001.zip › polymers-2552887-supplementary.pdf]

Article

Multi-Functional Ethylene-vinyl Acetate Copolymer Flexible Composite Film Embedded with Indium Acetate-Passivated Perovskite Quantum Dots

Sheng Huang *, Shasha Gao, Hui Zhang, Ce Bian, Yulong Zhao, Xiuquan Gu * and Wenjie Xu *

School of Materials Science and Physics, China University of Mining and Technology, Xuzhou 221116, China; sdyulong@cumt.edu.cn (Y.Z.)

* Correspondence: huangsheng@cumt.edu.cn (S.H.); xqgu@cumt.edu.cn (X.G.); ts21180100p31@cumt.edu.cn (W.X.)

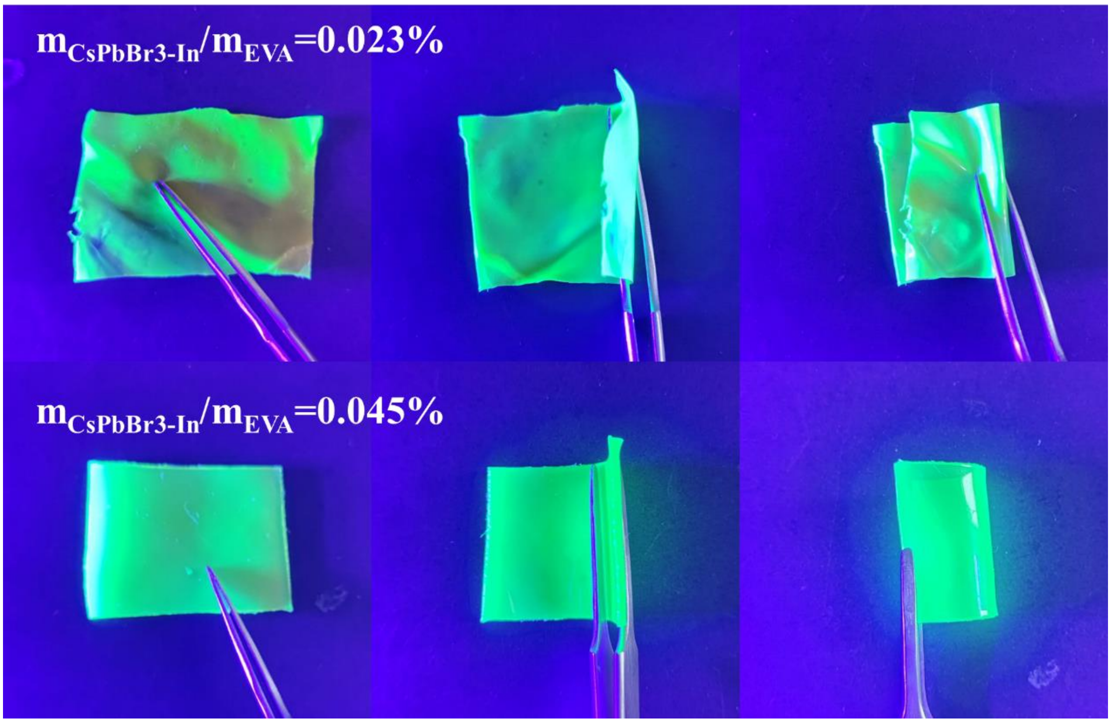

Figure S1. Bending test of different mass ratios of QDs and EVA.

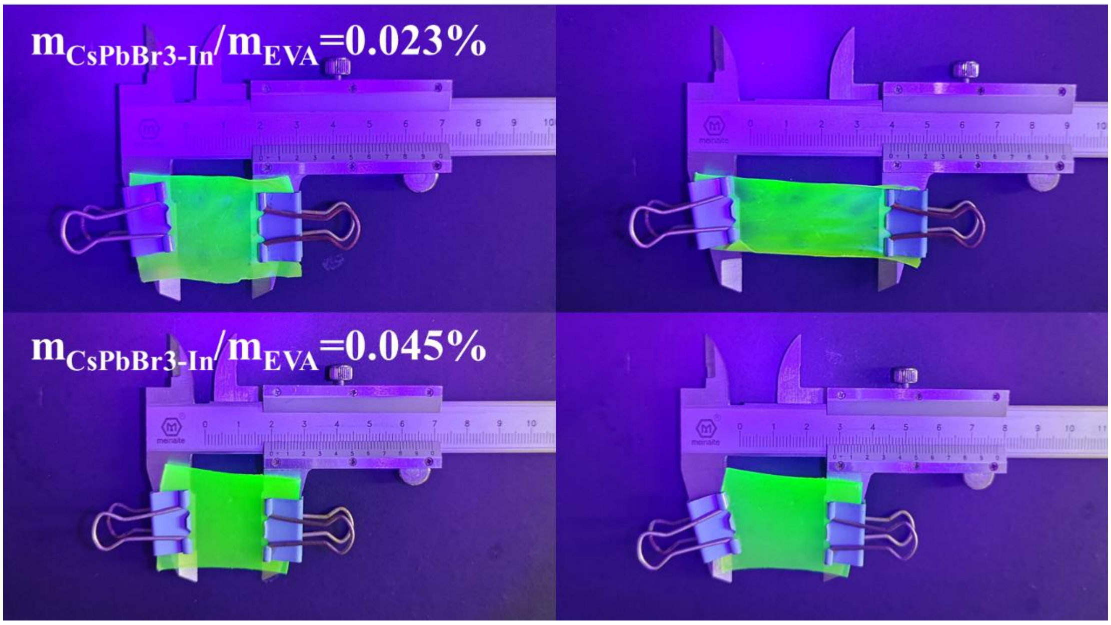

Figure S2. Stretch test of different mass ratios of QDs and EVA.

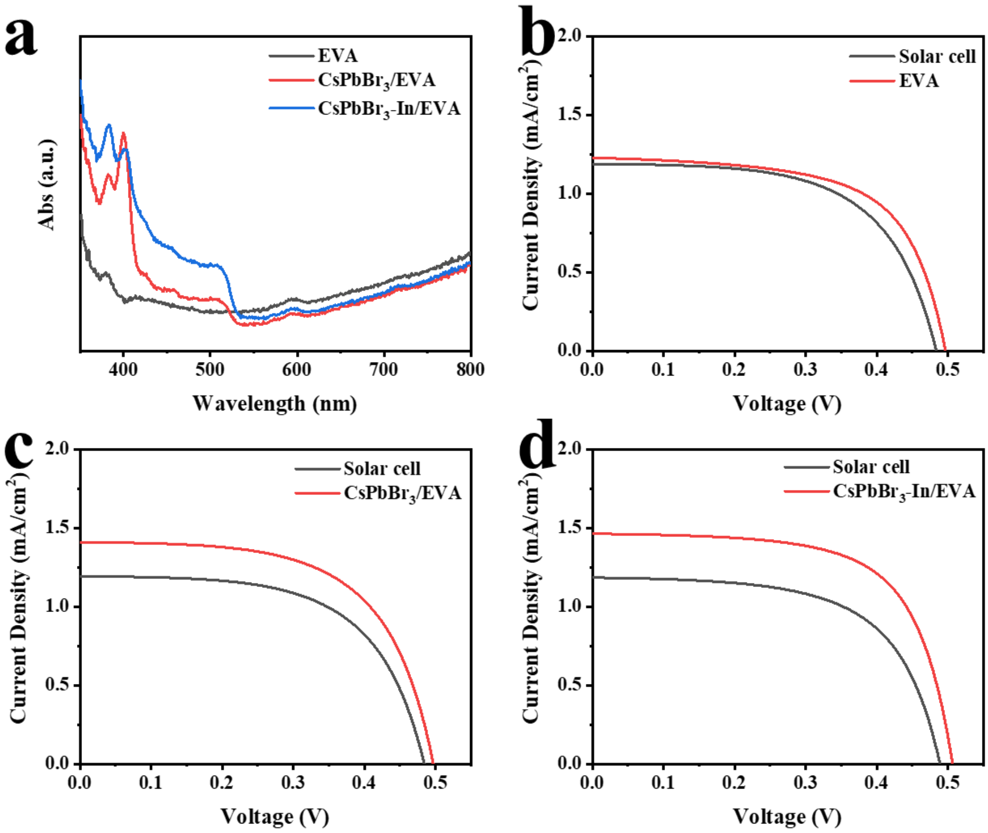

Figure S3. (a) UV-Vis spectra of EVA, $CsPbBr_3$/EVA and $CsPbBr_3$-In/EVA. J-V curves of silicon solar cells using (b) EVA, (c) $CsPbBr_3$/EVA, (d) $CsPbBr_3$-In/EVA under UV lights.

Table S1. Photovoltaic parameters of devices with EVA, $CsPbBr_3$/EVA and $CsPbBr_3$-In/EVA compo-site films under UV lights.

Figure	Sample	Voc (V)	J_{SC} (mA/cm^2)	Factor	Efficiency	ΔE
b	Solar cell	0.48322162	1.18983530	60.1394	0.3458	0.0348
	EVA	0.49635451	1.22934720	62.3665	0.3806	
c	Solar cell	0.48385300	1.19463180	60.2808	0.3484	0.0773
	$CsPbBr_3$/EVA	0.49657827	1.41100694	60.7529	0.4257	
d	Solar cell	0.48849077	1.18563882	61.2559	0.3548	0.1301
	$CsPbBr_3$ - In/EVA	0.50638190	1.46464626	65.3731	0.4849	
